# Sustained Effects of Developmental Exposure to Ethanol on Zebrafish Anxiety-Like Behaviour

**DOI:** 10.1371/journal.pone.0148425

**Published:** 2016-02-10

**Authors:** Matteo Baiamonte, Matthew O. Parker, Gavin P. Vinson, Caroline H. Brennan

**Affiliations:** 1 School of Biological and Chemical Sciences, Queen Mary University of London, London, United Kingdom; 2 School of Health Sciences and Social Work, University of Portsmouth, Portsmouth, United Kingdom; University Zürich, SWITZERLAND

## Abstract

In zebrafish developmentally exposed to ambient ethanol (20mM-50mM) 1–9 days post fertilization (dpf), the cortisol response to stress has been shown to be significantly attenuated in larvae, juveniles and 6 month old adults. These data are somewhat at variance with similar studies in mammals, which often show heightened stress responses. To test whether these cortisol data correlate with behavioural changes in treated animals, anxiety-like behaviour of zebrafish larvae (9dpf and 10dpf) and juveniles (23dpf) was tested in locomotor assays designed to this end. In open field tests treated animals were more exploratory, spending significantly less time at the periphery of the arena. Behavioural effects of developmental exposure to ethanol were sustained in 6-month-old adults, as judged by assessment of thigmotaxis, novel tank diving and scototaxis. Like larvae and juveniles, developmentally treated adults were generally more exploratory, and spent less time at the periphery of the arena in thigmotaxis tests, less time at the bottom of the tank in the novel tank diving tests, and less time in the dark area in scototaxis tests. The conclusion that ethanol-exposed animals showed less anxiety-like behaviour was validated by comparison with the effects of diazepam treatment, which in thigmotaxis and novel tank diving tests had similar effects to ethanol pretreatment. There is thus a possible link between the hypophyseal-pituitary-interrenal axis and the behavioural actions of developmental ethanol exposure. The mechanisms require further elucidation.

## Introduction

The damaging effects of ethanol exposure during development in humans have been amply described, and its subsequent behavioural consequences, are part of a range of symptoms collectively known as Fetal Alcohol Spectrum Disorder [1]. Some of these symptoms, like growth and facial defects, are apparent at an early age [2,3]. Others become clear in later life, for example children from alcoholic mothers are more likely to become drug addicts in adolescence or in adulthood, and may develop personality and psychotic disorders [4,5].

The mechanisms underlying such sustained effects of prenatal ethanol exposure in humans are still obscure, but it is now widely believed that the hypophyseal–pituitary–adrenal (HPA) axis is involved [6,7] since prenatal ethanol exposure frequently results in increased HPA tone and heightened HPA responsiveness in infancy which persists through adolescence into adulthood [6,8,9]. In rats too, animals prenatally exposed to ethanol show differences in HPA tone, revealed by elevated plasma ACTH and corticosterone levels in response to acute and chronic stressors.[10–15]. Additionally, both in humans and rats developmental exposure to ethanol evokes changes in behavioural measures of stress-reactivity in later life [16–24].

Effects of ethanol on the HPA axis are not only seen during development. In post-natally treated animals the HPA is invariably perturbed by alcohol, with severity depending on several factors including age of the animal, dose and duration of exposure [25–27]. These and other studies have led to the widely accepted hypothesis that prenatal ethanol exposure induces long lasting adaptation at multiple levels within the HPA axis, resulting in alterations in both HPA drive and feedback regulation [17,28,29]. All of this raises many interesting questions, most notably what is the precise nature of the link between developmental alcohol exposure and the HPA?

It is clear that animal models replicate many of the human findings [30–32]. While much value has been obtained from the study of various mammals, mostly rodents, others have sought answers in the zebrafish, in which tractability and transparency, conferring relative ease of use, are key advantages [33–37]. However, in marked contrast to the previous studies in humans and mammalian models, in zebrafish it was found that after developmental exposure to ambient ethanol (20mM-50mM) 1–9 days post fertilization (dpf), the cortisol response to stress was not heightened but significantly attenuated in larvae, juveniles and 6 month old adults [38]. Accordingly, it is appropriate to determine whether this unexpected finding of a decreased cortisol response to stress is accompanied with reduced anxiety-like behaviour, as the concept of a linkage between the HPA and behaviour would suggest.

Several behavioural measures have been developed to assess stress or “anxiety” levels in zebrafish, including thigmotaxis (time spent at the edge of apparatus), scototaxis (time spent at the bright side of tanks), “freezing” and novel tank diving (time spent at the bottom of tanks) [39–41], as well as tests for social interaction, or shoaling. Here we exploit established assays thought to reflect stress-related responses, to determine whether the clear effects of developmental ethanol exposure on the cortisol response of the HPI are associated with changes in behaviour.

## Materials and Methods

### Animal maintenance

All animal work was carried out following approval from the Queen Mary Research Ethics Committee, and under license from the Animals (Scientific Procedures) Act 1986. Care was taken to minimize the numbers of animals used in this experiment in accordance with the ARRIVE guidelines (http://www.nc3rs.org.uk/page.asp?id=1357). All behavioural experiments were carried out with systematic variation and randomization of housing allocation by treatment, as suggested by Parker [42]. Where relevant animals were sacrificed using terminal anaethesia with tricaine or other approved anaesthetic.

Zebrafish (*Danio rerio*) (Tuebingen wild type) were kept on a constant 14h:10h light:dark cycle at 28°C and fed 3 times a day with flake food and brine shrimp. All fish were bred and reared in the aquarium facility at Queen Mary University of London, licensed by the UK Home Office. Fish water used was prepared by dissolving sodium bicarbonate (0.9mM), calcium sulphate (0.05mM) and marine salts (0.018g/l)(Sigma, Poole, UK).

Embryos were separated from unfertilized ova and selected at the 8-cell stage to minimise age differences. For further accuracy embryos were staged using head-trunk angle and the optic vesicle length at 24 hours post fertilisation (hpf) [43]. They were then grouped in Petri dishes (Sterilin, Newport Gwent, UK) containing 50 embryos in 40ml of fish water for each treatment (ethanol or control) and reared in an incubator set at 28°. Embryos were collected and treated on 3 separate days.

Larvae were fed with Zmsystems ZM-000 high protein food particles (Tecniplast UK, London) from 5dpf-10dpf, ZM-100 and paramecium from 11dpf-14dpf, and ZM-200 and brineshrimp from 14dpf-30dpf. At one month of age, animals were transferred into aquaria where they were fed ZMsystems flake food and brineshrimp. For developmental ethanol exposure, treated larvae were exposed from 1–9dpf to 20mM or 50mM GPR ethanol (VWR, Lutterworth UK). Controls were handled similarly, but ethanol was omitted. For each experiment, control and experimental larvae were age and size matched: adults were sex matched in addition.

### Image collection

For zebrafish larval and juvenile thigmotaxis assays, a high-throughput imaging system for automated analysis was used. This comprises a 15-megapixel infrared Imaginsource digital camera DMK21AF04 attached to the lower shelf of an acrylic cabinet to allow filming from below the testing plate placed on the upper shelf. Either a 12-well plate (for 9dpf and 10dpf larvae) or a 6-well plate (for 23dpf juveniles) was used for this assay.

For the automated analysis of zebrafish adult behaviour a 15-megapixel SONY digital camera was used to film from above the tank, except in the case of the novel tank diving assay in which filming was from the side. Captured footage was automatically analysed using EthoVision XT 10 (Noldus, Wageningen).

### Thigmotaxis

#### Larvae and juveniles

Methods followed those of Richendrfer et al [44]. Larvae or juveniles were raised in Petri dishes or nursery tanks. One hour prior to the assay, animals were transferred in 6-well plates by pipetting with no more than 12 larvae or 3 juveniles per well. Wells were filled with 10ml of fish water. Experimental animals were transferred by pipetting them individually to a well in a 12-well plate (22mm diameter, used for 9dpf larvae) or a 6-well plate (34mm diameter, used for 23dpf juveniles), and immediately placed on the recording apparatus as described above.

The automated software (EthoVision) analyses the time spent at the outer zone of the well in the 6-well plate. The outer zone was defined as the region 4mm (average body length of a 9dpf larva) or 8mm (average body length of a 23dpf juvenile) from the edge of the well. Larvae were filmed for 5–10min, and juveniles for 5min. For characterization of the effects of diazepam on thigmotaxis, larvae were pretreated with 0.1mg/l diazepam (cf. reference [44]) for 6 minutes prior to thigmotaxis assessment. Diazepam was present at the same concentration throughout the thigmotaxis assay.

#### Adults

Experimental and control adults were housed in adjacent compartments of the same tank for 2 weeks prior to the start of the assay. Thigmotaxis was assayed using white opaque polypropylene circular tanks (410mm height x 320mm diameter) filled with 2l of fish water. The outer zone was defined as the region 4cm (the average length of an adult fish) from the edge of the tank, and the time spent in this zone was determined.

Tests were performed during the light phase at least 2 hours after lights on and feeding, between the hours of 11.00am and 5.00pm. Animals were carefully transferred into the tanks using hand-nets and immediately filmed for 6 minutes. Ethanol treated and control groups were tested alternately in four identical tanks. For characterization of the effects of diazepam on adult thigmotaxis, zebrafish were pre-incubated in 5mg/l diazepam (cf reference [45]) for 6 minutes prior to thigmotaxis assessment. Diazepam was present at the same concentration throughout the thigmotaxis assay.

### Novel tank diving

Methods followed those of Parker et al. [37]. Experimental and control adults were housed in adjacent compartments of the same tank, for 2 weeks prior to the start of the assay. Novel tank diving was then assayed using trapezoid tanks (152mm height, 279mm top length/225mm bottom length, 71mm width) filled with 1.5l fish water.

Tests were performed during the light phase (9am-5pm). Animals were transferred to the novel tanks using hand-nets and immediately filmed for 5 minutes. Ethanol treated and control groups were tested alternately in identical tanks. Diving was assessed as the time spent in the lower third of the tank (approx. 50mm). For characterization of the effects of diazepam on novel tank diving, zebrafish were pre-incubated in 5mg/l diazepam (cf reference [45]) for 6 minutes prior to transfer to the novel tank environment. Diazepam was present at the same concentration throughout the novel tank diving assay.

### Scototaxis

Methods followed those of [46]. Experimental and control adults were housed in adjacent compartments of the same tank for 2 weeks prior to the start of the assay. White opaque tanks (330mm length x 160mm width x 130mm height) containing 2l fish water were used. They were divided into two compartments by a black opaque acrylic divider, with a square hole in the middle (50 x 50mm). One side of the tank was exposed to light and the other side was darkened.

Animals were transferred using hand-nets into the lit side of the tanks and immediately filmed for 9 minutes on the lit side. Treated and control groups were tested alternately in identical tanks.

### Statistics

Stress reactivity data were fitted to a linear mixed effects model [47] with fixed effects that included ‘ethanol dose’ and ‘time’ using R software (R Development Team). ‘Fish ID’ nested in ‘housing tank’ was included as a random effect. Distance travelled was entered as a covariate in all models to account for immobility and darting periods. The dependent variables were the period of time spent in the designated zones. Post-hoc Student’s T-tests were applied to characterise simple main effects and interactions.

## Results

### Thigmotaxis

#### Larvae and juveniles

**Fig 1:**
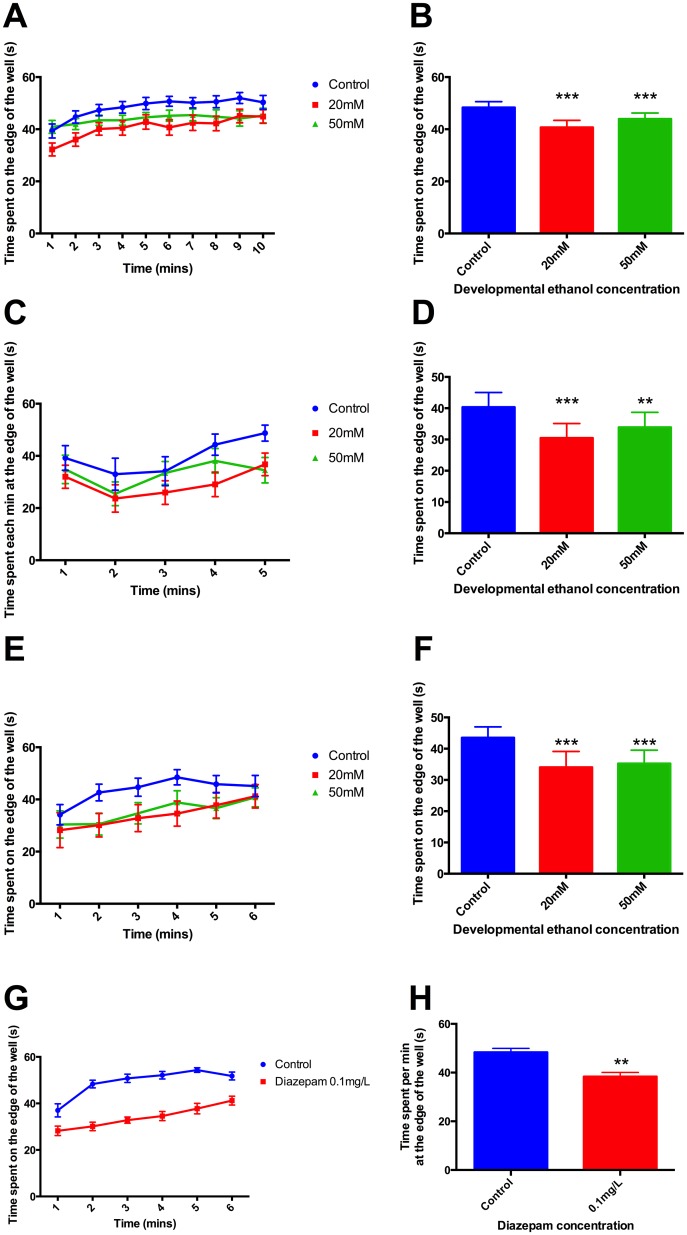
Stress-related behaviour assessed by thigmotaxis in zebrafish larvae A,B) 9dpf, C,D) 10dpf, E,F) 23dpf juveniles. G,H) Effect of diazepam on larval stress-reactivity assessed by thigmotaxis. Time course of average time spent each minute at the edge of the apparatus (A, C, E), overall average time spent per minute at the edge of the apparatus (B, D, F). (A-D) Developmental ethanol exposure decreased thigmotaxis at both 9dpf (A,B: *F* 2,105 = 4.76, *P*<0.05) and 10dpf (C,D: *F* 2, 285 = 6.69, *P*<0.05), with the greatest difference between 20mM ethanol treatment and the control. Siblings of the same animals were raised for another 2 weeks and tested as 23 dpf juveniles (E,F). These juveniles exhibited a similar thigmotaxis response as at 9dpf, with decreased thigmotaxis in ethanol treated animals compared to controls (*F* 2,146 = 2.93, *P*<0.05). (G-H) Larvae acutely treated with diazepam for 6 minutes exhibited significantly reduced time spent at the edges of the wells compared to controls (*F* 1, 259 = 5.47, *P*<0.01). There were no significant differences in distance travelled. Post-hoc t-test: *** *P*<0.001, ** *P*<0.01.

Control and treated animals were tested at 9dpf, 10dpf and 23dpf stages for differences in thigmotaxis, thus during ethanol treatment, and, their siblings, at 10dpf and 23dpf, one day or two weeks after treatment had ended. Decreased time spent at the periphery of the wells was observed at all stages (Fig 1), although there was no obvious dose relationship at the two concentrations of ethanol used. Zebrafish 9dpf larvae acutely exposed to diazepam (0.1mg/L) exhibited reduced time spent at the edge of wells (Fig 1G and 1H). There were no differences between the groups in the distances travelled. Experiments were repeated on 3 separate occasions using approximately 50 animals in each treatment group on each occasion

#### Adults

**Fig 2:**
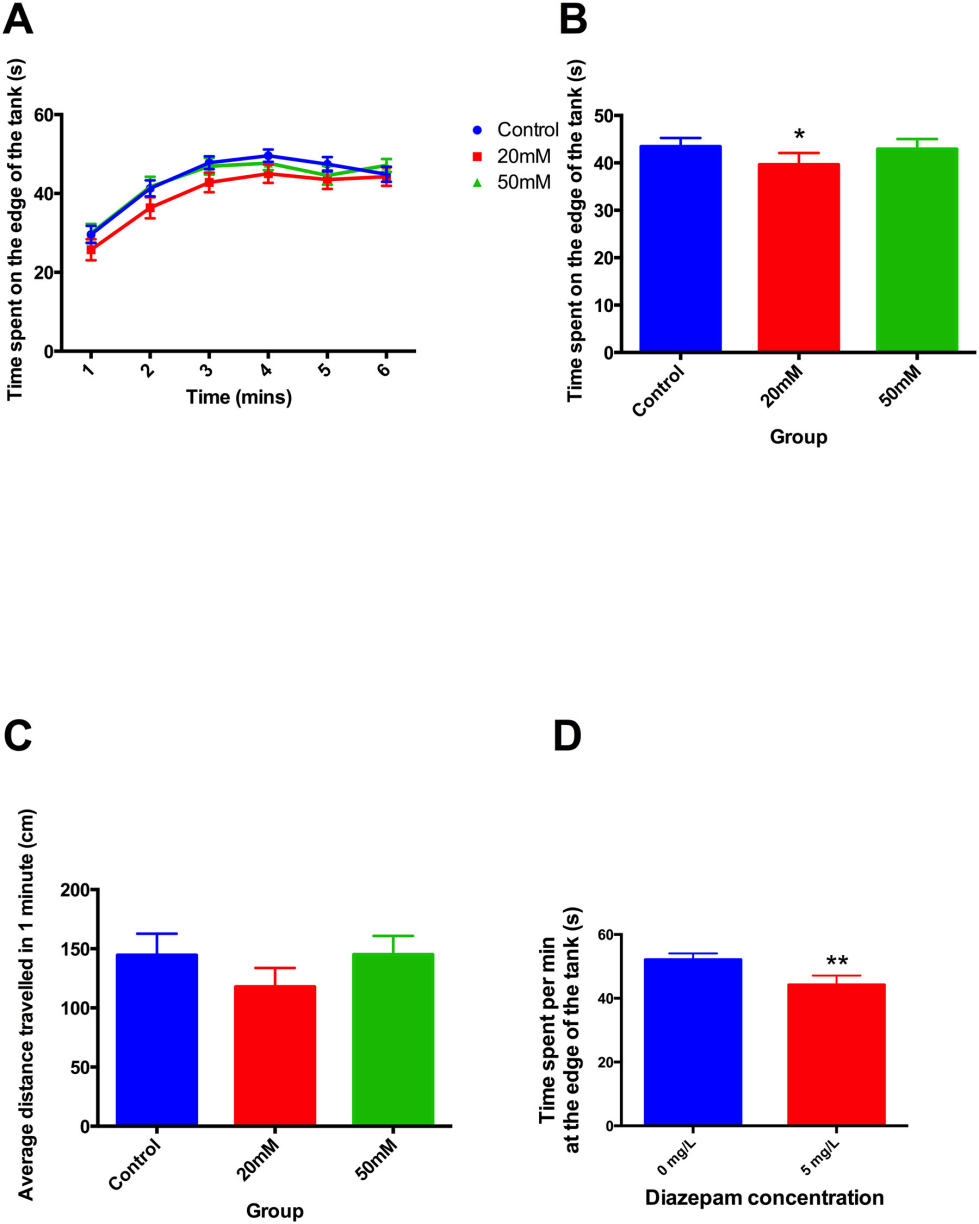
Stress-reactivity of 6-month old zebrafish as assessed by thigmotaxis. **A)** Time course of average time spent per minute at the edge of the apparatus, **B)** overall average time spent per minute at the edge of the apparatus, **C)** distance travelled during thigmotaxis, **D)** Effect of diazepam on adult zebrafish stress-reactivity assessed by thigmotaxis. **A,B**) Adult zebrafish that had been experimentally exposed to ethanol spent decreased time at the edge of the tank, (*F* 2, 127 = 3.09, *P*<0.05). There were no significant differences in distance travelled (C). Adults acutely exposed to diazepam exhibited reduced time spent at the edges of the tanks compared to controls (*F* 1, 4.98 = 5.44, *P*<0.001) (D). Post-hoc t-test, * *P*<0.05.

Adult zebrafish that had been treated developmentally with 20mM ethanol exhibited a decrease in time spent at the edge of the circular tank compared to controls (Fig 2), however, animals treated with 50mM ethanol showed no change. Zebrafish 6-month old adults acutely exposed to diazepam (5mg/L) exhibited reduced time spent at the edge of the tanks (Fig 2D). There were no differences between the groups in the distances travelled. Experiments were repeated on 6 separate occasions using approximately 20 animals in each treatment group on each occasion

### Novel tank diving

**Fig 3:**
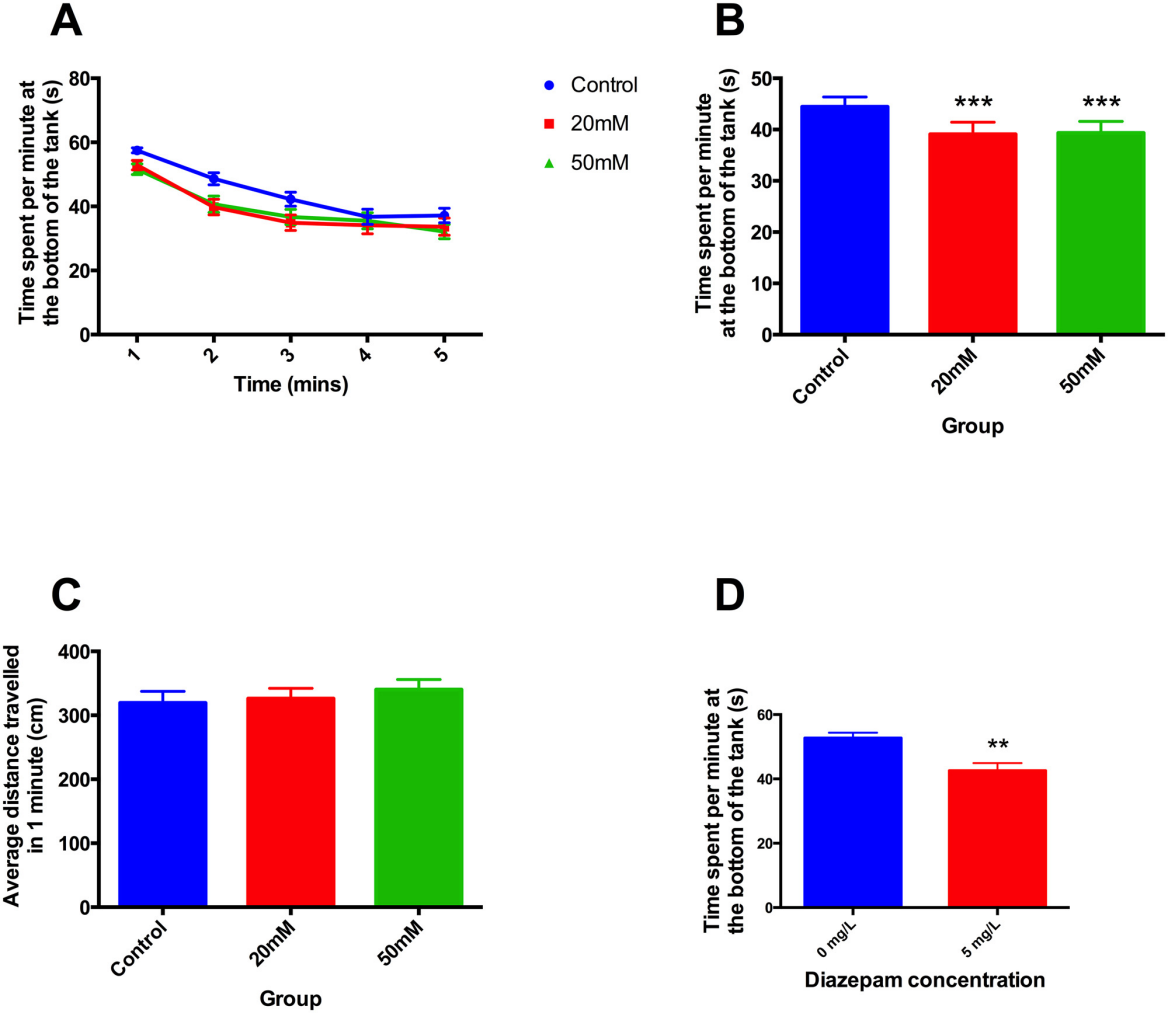
Stress-reactivity measured by novel tank diving in 6-month old adult zebrafish. **A)** Time course of average time spent per minute at the bottom of the tank, **B)** overall average time spent per minute at the bottom of the tank each minute, **C)** mean distance travelled per minute during novel tank diving. **D)** Effect of diazepam on zebrafish stress-reactivity assessed by novel tank diving. A,B. Zebrafish that had been developmentally exposed to ethanol showed reduced bottom dwelling (*F* 2, 682 = 3.47, *P*<0.05). There were no significant differences in distance travelled (C). D. Diazepam also significantly reduced time spent by adults at the bottom of the tanks compared to controls (*F* 1, 408 = 5.45, *P*<0.001). Post hoc t-test, *** *P*<0.001.

Adults treated with ethanol during development exhibited decreased bottom dwelling in the novel tank. There were no differences between the groups in the distances travelled. (Fig 3C). Zebrafish 6-month old adults acutely exposed to diazepam (5mg/L) exhibited reduced time spent at the bottom of the tanks (Fig 3D). Experiments were repeated on 3 separate occasions using approximately 20 animals in each treatment group on each occasion

### Scototaxis

**Fig 4:**
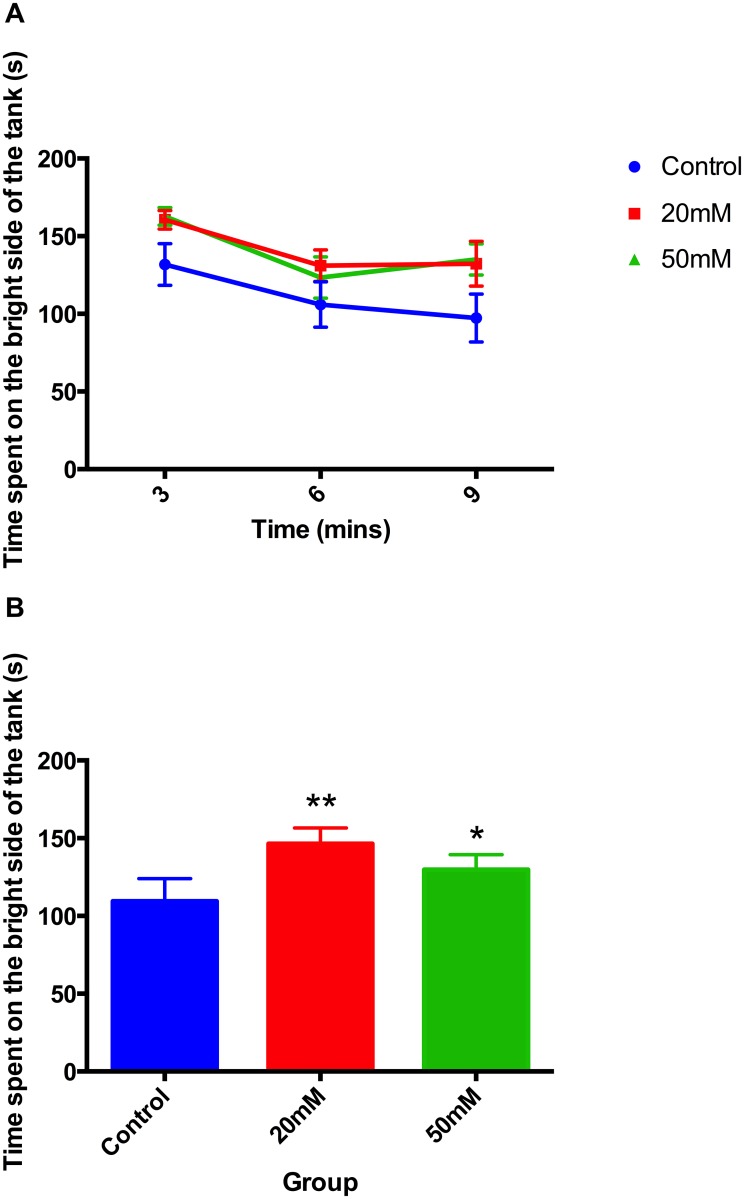
Stress-reactivity measured by scototaxis in 6-month old adult zebrafish A) Time course of average time spent at the bright side of the apparatus and B) overall average time spent at the bright side of the apparatus. Adult zebrafish that had been developmentally exposed to ethanol spent more time on the bright side of the tank (*F* 2, 31 = 3.85, *P*<0.05). Post hoc t-test, ** *P*<0.01, * *P*<0.05.

Early ethanol exposure caused an increase in time spent on the bright side of the tank compared to controls (Fig 4). This effect was more prominent with animals that had been developmentally exposed to 20mM ethanol. Due to assay limitations, animal tracking in the dark side of the tanks was not recorded; therefore distance travelled for scototaxis was not assessed. Experiments were repeated on 3 separate occasions using approximately 10 animals in each treatment group on each occasion

## Discussion

The HPA axis, and extrahypothalamic CRH, are thought to be involved in adult responses to both acute and chronic ethanol exposure, and show characteristic responses to ethanol and other substance abuse in humans and in mammalian models [48–50], and also in zebrafish [33–37]. We recently demonstrated that developmental exposure to ethanol in zebrafish causes a sustained effect on whole body cortisol. In these experiments, the cortisol response to stress was dampened in both larvae and juveniles 1 day and 2 weeks after exposure, and also in adults, 6 months after treatment [38].

It is now clear from the present results that this change in cortisol response is accompanied by behavioural changes. The behavioural characteristics studied here are often interpreted as measures of fear or anxiety. Thus animals that are threatened by new surroundings show characteristic responses in thigmotaxis, scototaxis and tank diving that can be interpreted as strategies to minimise detection. In general, the ethanol-evoked changes shown in Figs 1–4 in thigmotaxis, scototaxis and novel tank diving, show exploratory movement indicating relative absence of stress, which fits well with the lower response to stress in the cortisol data [38]. This interpretation is supported by data shown in Figs 1–3 indicating diazepam treatment has a similar effect to reduce thigmotaxis, and tank diving as evoked by developmental ethanol exposure (Figs 1–4).

The association between these two sets of data, cortisol and behavioural does not necessarily imply causality. It is nevertheless striking how often these are linked.

Stress is a complex concept. It is impossible to quantitate, except through secondary indicators. Of these, cortisol is perhaps the most frequently used (or corticosterone in rats or mice). Caution must be exercised however, because its actual function in the response to stress remains obscure, and its use as a measure of stress is confounded by its regulation by other factors not obviously stress-related, such as the time of day [51]. For this and other reasons it may be unwise to extrapolate from the human species (in which stress, though widespread, remains a subjective phenomenon, not measurable by observers) to the zebrafish whose physiological situation and demands are very different.

Nevertheless, the similarities between mammalian and zebrafish responses to stressful situations have been used to promote the view that the zebrafish can be used as a model for human emotional states such as anxiety, resulting from stress [40,41,52]. This gains credibility from the actions of drugs, especially known anxiolytics such as diazepam, on zebrafish behaviour in response to stress [53,54] c.f. Figs 1–3. It is also supported by relating behavioural stress responses to whole body cortisol [55,56].

Using this interpretation then, the present results suggest that developmental ethanol exposure leads to a phenotype that is hyporeactive to stress, as evidenced by reduction in both behavioural measures of anxiety and cortisol levels. Going further, developmental exposure to ethanol not only appears to be anxiolytic, as judged by thigmotaxis in larvae and juveniles 1 day or 2 weeks following treatment, but also in adults some 6 months after the cessation of treatment as assessed by novel tank diving, thigmotaxis and scototaxis (Figs 1–4).

These data thus partly confirm but partly contrast with those of others. In mammals the behavioural and cortisol response to ethanol is complex. Acutely, ethanol stimulates corticosterone secretion in adult rats [25,57], but longer exposure is associated with depressed HPA activity [27]. Ethanol withdrawal produces anxiogenic symptoms, including elevated corticosteroid, in humans, rodents, and in zebrafish [27,58–62]. Furthermore, developmental exposure to ethanol in rodents leads in later life to similarly enhanced HPA responses to stress as in the human species [31,63]

More pertinent to the present studies are those of Fernandes and Gerlai [64] and Bailey et al [65] both of whom examined long term effects of early developmental ethanol exposure on subsequent adult behaviour in zebrafish. The periods of exposure were far shorter than those used in the present study, just 2h at 24hpf [64], and for 2h at 8hpf or 3h at 24hpf [65] and the concentrations of ethanol used were up to 1% (171mM) [64], or 1–3% [65], thus approximately 5–15 times higher than the maximal concentration (50mM) used here. Fernandes and Gerlai [64] used a shoaling assay, and showed that adult fish (6 months) that had been exposed to higher concentrations significantly distanced themselves from a computer animated zebrafish shoal. Bailey et al [65] tested their treated animals at 2 months, and found significant effects in a tap startle assay, and in novel tank diving. Both of these tests showed greater activity in the treated animals relative to the controls. Bailey et al [65] also conducted novel tank diving assays and showed that treated animals were more exploratory and spent less time at the bottom of the tank, consistent with the results reported here.

There may be differences in interpretation of all such behavioural data, but ours tends to support the view that 1–9 day ethanol treatment has sustained actions that are primarily anxiolytic in zebrafish, thus contrasting with rodent data do not reflect those in rodents in which prenatal ethanol subsequently produces symptoms of anxiety and depression [29,66]. Clearly differences in handling and procedure can give variations in results, and we have found that use of somewhat different treatment protocols may give some data at variance with that described here ([37] and unpublished).

As mentioned in the introduction, we opted to use ethanol doses that reflect ethanol concentrations experienced by mammalian fetuses during development, which yielded in an anxiolytic phenotype. We acknowledge that different ethanol doses could potentially yield a different phenotype under this same exposure period, as it has occurred with mice and zebrafish during different points of development [67,68].

The more important point that we seek to emphasise in this paper however, is that taking the present data together with that from our previous study [38], there is a clear relationship between behaviour and HPI function, reflecting similar results in mammals [50,58–60]. The mechanisms merit further investigation.

## Supporting Information

S1 Fig (XLSX)

S2 Fig (XLSX)

S3 Fig (XLSX)

S4 Fig (XLSX)
